# Lung Cancer through Transcription Factors

**DOI:** 10.3390/ijms24119461

**Published:** 2023-05-30

**Authors:** Kostas A. Papavassiliou, Nektarios Anagnostopoulos, Athanasios G. Papavassiliou

**Affiliations:** 1 First Department of Respiratory Medicine, ‘Sotiria’ Hospital, Medical School, National and Kapodistrian University of Athens, 11527 Athens, Greece; konpapav@med.uoa.gr (K.A.P.); aris.anag@yahoo.gr (N.A.); 2 Department of Biological Chemistry, Medical School, National and Kapodistrian University of Athens, 11527 Athens, Greece

The body of knowledge on the molecular mechanisms that drive lung cancer, including non-small cell lung cancer (NSCLC) and small cell lung cancer (SCLC), is continuously growing. Lung cancer research has revealed a substantial amount of information regarding the oncogenic signaling pathways that promote the pathobiology of NSCLC and SCLC. Yet, the transcriptional circuits that mediate these upstream signaling pathways and downstream functional effects are still largely unknown. Transcription factors are DNA-binding proteins located at the helm of multiple signal transduction cascades and are responsible for orchestrating gene expression programs that ultimately result in changes in cell behavior. Accumulating evidence suggests that mutated (including fused) or deregulated transcription factors are of paramount importance for the development and progression of lung cancer [1,2].

Transcription factors associated with NSCLC and SCLC have been shown to play a key role in proliferation, invasion, migration, tumor-associated immunity, drug resistance, trans-differentiation and many other malignant features [3,4,5,6,7,8,9]. Not only do transcription factors affect the pathobiology of lung cancers, but their expression also defines lung cancer subtypes. Recent data from primary human tumors, patient-derived xenografts, cancer cell lines and mouse models suggest the existence of distinct molecular subtypes of SCLC based on the relative expression of four transcription factors, namely yes-associated protein 1 (YAP1), neurogenic differentiation 1 (NeuroD1), achaete-scute family BHLH transcription factor homolog 1 (ASCL1) and POU class 2 homeobox 3 (POU2F3) [10].

Given the pivotal role of transcription factors in lung carcinogenesis and malignant progression, these proteins represent promising therapeutic targets [11]. Although there are many challenges that must be overcome with respect to transcription factor targeting, their pharmacological modulation may be a better strategy compared with current therapeutic regimens. Since transcription factors are situated at the convergence of oncogenic signaling and are crucial for aberrant gene expression, blocking their function will probably result in the successful inhibition of cancer cell hallmarks without cancer cells being able to easily develop bypass mechanisms of resistance. Transcription-factor-targeting drugs may also be combined with other therapies, such as immune checkpoint inhibitors, for the clinical management of lung cancer [12,13]. An important aspect of such a therapeutic strategy will be to stratify patients according to a transcription-factor-based molecular profile in order to yield clinical benefits. Hence, research must also focus on identifying transcription factors which can serve as predictors of response to transcription-factor-inhibitory drugs.

The list of transcription factors related to the pathobiology of lung cancers is continuously expanding, and with the help of bioinformatics and the application of machine learning to transcription factor datasets, we can generate lung cancer gene regulatory networks of transcription factors [14,15]. Automated wet lab experiments will validate the biological relevance of all of these transcription factors in lung cancer and will inform drug development processes.

Unraveling the molecular details of these transcriptional networks will help cancer investigators and clinical oncologists to better understand the malignant behavior of lung cancer. Mapping the interactions of transcription factors with each other and their cofactors (coactivators, corepressors, chromatin modulators) will move the field of lung cancer research forward to decipher the complexity and heterogeneity of NSCLC and SCLC, as well as provide insights that may be translated into effective strategies for the clinical management of lung cancer patients.
